# Origin of Sex-Biased Mental Disorders: Do Males and Females Experience Different Selective Regimes?

**DOI:** 10.1007/s00239-022-10072-2

**Published:** 2022-09-12

**Authors:** C. Michelle Brown, Queenie Wong, Aditi Thakur, Karun Singh, Rama S. Singh

**Affiliations:** 1 Department of Biology, McMaster University, Hamilton, Canada; 2 Krembil Research Institute, University Health Network and Faculty of Medicine, University of Toronto, Toronto, Canada

**Keywords:** Sex-biased diseases, Mental disorders, Sexual selection, Women’s health, Social stress

## Abstract

The origins of sex-biased differences in disease and health are of growing interest to both medical researchers and health professionals. Several major factors have been identified that affect sex differences in incidence of diseases and mental disorders. These are: sex chromosomes, sex hormones and female immunity, sexual selection and antagonistic evolution, and differential susceptibility of sexes to environmental factors. These factors work on different time scales and are not exclusive of each other. Recently, a combined Sexual Selection-Sex Hormones (SS-SH) Theory was presented as an evolutionary mechanism to explain sex-biased differences in diseases and mental disorders (Singh in J Mol Evol 89:195–213, 2021). In that paper disease prevalence trends were investigated, and non-sex-specific diseases were hypothesized to be more common in males than in females in general. They showed signs of exceptions to this trend with inflammatory diseases and stress-related mental disorders that were more common in females. We believe that the SS-SH theory requires the consideration of psycho-social stress (PSS) to explain the predominance of female-biased mental disorders and some other exceptions in their findings. Here we present a theory of sex-differential experience of PSS and provide quantitative support for the combined SS-SH-PSS Theory using age-standardized incidence rates (ASIRs) recording the levels of male- and female-bias in data obtained from different countries. The grand theory provides an evolutionary framework for explaining patterns of sex-biased trends in the prevalence of disease and health. Further exploration of women’s vulnerability to social factors may help to facilitate new treatments for female-biased diseases.

## Introduction

Large discrepancies in the prevalence of diseases between males and females are not surprising when related to sex-specific reproductive organs, such as ovarian cancer and erectile dysfunction (Vos et al. 2020). However, these discrepancies also manifest themselves in a wide range of diseases that are seemingly unrelated to reproductive differences between sexes. Females generally show a higher prevalence of autoimmune diseases (Voskuhl 2011; Ngo et al. 2014), thyroid cancer (Kilfoy et al. 2009), and certain psychiatric disorders, like anxiety (McLean et al. 2011) and depression (Salk et al. 2017), while males show a higher prevalence of cancer (Bray et al. 2018) and infections. The sexual selection-sex hormone theory (SS-SH theory) was developed to explain sex-specific variability in disease incidence, which laid out the framework for differentiating between the historical (evolutionary or ultimate) and immediate (functional or proximal) causes of sex-related differences in diseases and mental disorders (Singh et al. 2021). Evolutionary and functional causes should not be considered an “either-or” scenario but instead as being evolved and hierarchically embedded. Furthermore, it has been noted that while the SS-SH theory can be used to explain the generally lower rates of cancer and higher rates of autoimmune diseases in females, the theory was not able to justify the higher rate of certain mental disorders in females that have been shown to exhibit sensitivity to stress (McLean et al. 2011; Salk et al. 2017). Presently, there is no general theory to explain the higher stress-related incidence of female mental disorders and researchers often overlook the vital interactions between society, stress, and disease.

In this commentary, we briefly summarize the sex- and sexual selection-driven causes of sex-biased diseases and mental disorders and then elaborate on the role of environmental factors. We present a theory, Psycho-Social Stress Theory (PSS Theory), to test the hypothesis that males and females experience different social stress. We lay out three separate mechanisms based on biological, psychological, and environmental exposure to PSS that could be contributing factors to sex-biased diseases and mental disorders.

We present results from worldwide disease incidence rates and the data show, on the face value, that in the long term, sexual selection may act as a major cause of sex differences in many diseases and mental disorders. For non-sex-specific diseases, females appear to be biologically protected but potentially socially vulnerable giving rise to high incidence rates of some mental illnesses that primarily affect females. We present a comprehensive Sexual Selection-Sex Hormone-Psycho-Social Stress Theory (SS-SH-PSS Theory) and explain how women’s health is affected, directly or indirectly, by sexual selection. We propose that, while not ignoring the fundamental biological and psychological causes, elevated levels of exposure to psycho-social stress should be investigated as a possible root cause of heightened stress-related mental disorders in females.

## An Overview of Sexual Selection and Sexual Antagonism

A sexual selection theory applicable to humans has been developed wherein sex is shown to have important consequences for female fitness (Singh and Kulathinal 2005; Jagadeeshan et al. 2015; Singh and Jagadeeshan 2020), and some have successfully applied the theory to the evolution of human menopause (Morton et al. 2013; Takahashi et al. 2017; Chan et al. 2020) and maternal mortality (Jagadeeshan et al. 2019).

For a recent and comprehensive outline of the sexual selection theory and how it may lead to the evolution of sex-biased diseases and mental disorders, the reader is referred to Singh et al. (2021). In this section, we provide a brief outline of sexual selection and antagonistic evolution, as a means of transitioning the discussion from sexual selection and sex hormones to the role of sexual differences in the experience of stress and its effect on sexual dimorphism in disease and health.

### Darwin’s Sexual Selection Theory

Charles Darwin’s canonical sexual selection theory consists of two components: direct intra-male competition, in which males attempt to outcompete their rivals to win mating opportunities with females; and female choice, whereby females choose their mates based on personal preference (Parker 1979). Males and females employ different reproductive strategies; these often result in dissimilar ideal or optimum traits between the sexes. Males have higher reproductive variability, and, in most cases, because of their small gamete size, they contribute fewer resources to the offspring than females (Bateman 1948). Due to the high costs of reproduction, females are usually only able to produce a limited number of offspring at any one time and, therefore, have a more consistent rate of reproduction. Males, on the other hand, often need to make considerably less of a commitment and can reproduce by mating with a virtually unlimited number of females. As a result, sexual selection plays a key role in determining which males pass on their genes, and which do not. As is to be expected, the consequences of sexual selection are more profound for males, including a greater overall effect on their fitness (Bateman 1948; Trivers 1972).

These opposing male/female priorities create complications in the evolutionary drive for higher fitness levels and prompt situations to which the sexes respond. A mutation that benefits one sex may be deleterious for the other, preventing that mutation from becoming fixed in the population (Van Doorn 2009; Pennell and Morrow 2013), or prompting responses to it through counter-mutations to suppress the deleterious effects (Bonduriansky and Chenoweth 2009; Connallon and Clark 2014). As such, antagonistic evolution could be a cause of the sex bias demonstrated in non-sexual diseases in humans (McKean and Nunney 2005).

Singh et al.’s (2021) SS–SH theory considers a combination of male-driven sexual selection and sex hormone fluctuation during the reproductive years of life, and the effect of that combination in terms of female vulnerability to diseases such as migraines and depression (Brummelte and Galea 2016; Artero-Morales et al. 2018; Slavich and Sacher 2019). The combined theory posits that the effects of sexual selection are responsible for the prevalence of certain diseases in males, and that female sex hormones, in combination with certain social conditions, are responsible for the high incidence of other diseases in females. The SS-SH theory, however, did not provide details as to how sexual selection is likely to be linked to males’ and females’ experiences, perceptions, or susceptibility of stress. In the present paper, we build on this physiological and psychological connection and show the relationship between hormones, immunity, and susceptibility to social stress.

## The Interactions Between Sex, Stress, and the Immune System

### The Relationship Between Stress and Inflammation

Both microscopic stress (on a cellular level) and macroscopic stress (on an individual level) play important roles in activating different systems in the body in response to certain stimuli. Within cells, stress causes cascades of enzyme reactions to enable an appropriate responsive measure to that specific stressor. Similar processes occur on an organism level, but hormones, instead of intracellular enzymes, act in a cascading effect to address any stress that can affect the whole organ or the organism. In vertebrates, this complex neuroendocrine system is described as the hypothalamus–pituitary–adrenal (HPA) axis (Denver 2009). Through the release of corticotropin-releasing factor (CRF), organisms change their physiology and behavior to keep themselves safe and healthy (Denver 2009). On a short-term basis, stress is incredibly beneficial, upregulating both the innate and adaptive immune systems (Dhabhar 2014).

However, stress begins to cause problems when it becomes chronic. Long-term exposure to stress results in immune dysregulation and low-grade chronic inflammation (Dhabhar 2014). In the twenty-first century, most people report experiencing high levels of stress throughout a large portion of their lives (World Health Organization 2001). One unfortunate result of this trend has been an increased predominance of mental distress and mental illness.

PSS and immune dysfunction have a tight-knit relationship. Stress causes changes that upregulate some immune pathways and downregulate others. Exposure to PSS is found to result in a general increase in activation of T cells from inflammatory cytokines (Schmidt et al. 2010). But the specific kinds and function of the T cells differ depending on the study (Schmidt et al. 2010, 2016; Kim et al. 2012). Additionally, experiences of childhood trauma have been positively linked to inflammatory cytokines such as TNF-⍺ and IL-6 (Baumeister et al. 2016).

Many investigations into stress exposure also find positive relationships between mental illnesses and inflammation. In general, depressed patients experience higher levels of pro-inflammatory cytokines with less variation than the average population (Dowlati et al. 2010; Osimo et al. 2020). Pro-inflammatory cytokines also appear to be inflated in individuals with generalized anxiety disorder (Costello et al. 2019; Uzun and Akıncı 2021), bipolar disorder (Solmi et al. 2021), panic disorder (Liu et al. 2021), post-traumatic stress disorder, and obsessive compulsive disorder (Renna et al. 2018), but there is less of a clear trend here with more contradicting findings within the literature than with depression.

Experiences of stress are likely also associated with autoimmune disorders. Higher levels of psychologically traumatic stress were found in patients with multiple sclerosis (Mohr et al. 2004), rheumatic diseases (Salihoglu et al. 2019), systematic lupus erythematosus (Roberts et al. 2017), psoriasis (Simonic et al. 2010; Wintermann et al. 2022), and irritable bowel syndrome (IBS; Bradford et al., 2012) than control groups.

### Sex Differences in the Immune System

The evolutionary mechanisms that are responsible for the sex-specific difference in immunity are complicated and result from a combination of many factors. Sex differences in immunosuppressive capacity could arise from the prioritization of fitness for reproductive selection. Since female fitness benefits from longevity, it is likely that the female body also invests more in immune functions (Rolff 2002). This investment could explain the increased immune reactivity in females and their greater resilience against many various types of disease. It is also possible that this investment is the factor that led to the evolution of making females more prone to developing autoimmune diseases (Ngo et al. 2014). In comparison to females, males tend to have a higher level of immune suppression; this is linked to the prioritization of male reproduction. The specific biological mechanisms for how this happen are likely complex and work through various bodily systems.

Current research has uncovered significant differences between male and female physiology, although there is still much to discover. One of the most obvious factors affecting incidence of genetic diseases between men and women are the sex chromosomes (X–Y). With two X chromosomes, females have more protection from deleterious, recessive X-linked traits; males on the other hand, with one X chromosome, are exposed to X-linked recessive traits. As a result, the incidence of X-linked diseases is much higher in males than in females (McKusick 1998). Considering that many immune-related genes exist on the X chromosome, it’s likely that any recessive mutation would affect males more than females (Schurz et al. 2019). This may explain some of the physiological mechanisms of immune differences between sexes.

Additionally, in females, one of the two X chromosome copies was once thought to be silenced to regulate the dosage of X chromosome-genes between sexes, but recent findings indicate otherwise (Gartler and Riggs 1983; Berletch et al. 2010). It appears that some genes may avoid inactivation, leading to a higher dosage of these genes in females than in males (Bianchi et al. 2012) and given the presence of immune genes within the X chromosome, this may further explain sex differences in immune response (Libert et al. 2010).

Molecular and genetic expression sometimes show interesting sex specificity that may explain some physiological differences in males and females. Firstly, some traits can vary in sex-specific manner (sex-specific heritability). For example, testosterone is a circulating hormone in both males and females but one study showed 119 female and 445 male-specific variants in the gene (Flynn et al. 2021). Additionally, evidence shows that gene expression can also differ by sex. Likely through differences in transcription factor binding, approximately 37% of genes in some tissues exhibit sex differences in expression (Oliva et al. 2020). These differences tend to be small, but even small differences over such a broad space can result in a noticeable difference. Finally, gene expression on larger cell levels can also show sex-specific differences. Belonwu et al. (2022) found that neurons in Alzheimer’s patients showed patterns of separations in males and females, particularly pre-frontal glial cells. Needless to say, these cellular and genetic sex differences don’t serve to invalidate the evolutionary origin of sex-biased diseases but serve as an explanation as to how exactly these evolutionary pressures have acted on the body to create physiological differences.

Sex hormones also have significant influences on many aspects of human biology. Testosterone and estrogen are known to modulate immune functioning, although the specific details of what the effects might be are still unclear (Foo et al. 2017). Testosterone is likely to suppress immune functioning, as is anticipated by the SS-SH theory (Singh et al. 2021). Estrogen, on the other hand, seems to increase certain immune functions and decrease others, depending on the dose, the sensitivity of the individual, the type of cell, the specific estrogen receptor present, and other factors (Khan et al. 2012). This hormone can have such extreme effects as upregulating the cytokines associated with the development of some auto-immune diseases, like lupus (Kassi and Moutsatsou 2010), and offer a potential treatment method for others by, for example, reducing inflammatory brain lesions in patients with multiple sclerosis (Gold and Voskuhl 2009).

Pregnancy causes intricate relationships between the host and fetus’ immune systems. Mothers’ immune systems are heightened in some ways to protect themselves and their babies from disease, but also downregulated in order to prevent the host’s immune system from attacking the fetus (Abu-Raya et al. 2020). Although the immune system is influenced by pregnancy, there is no evidence to support that immune changes during pregnancy cause increase immune reactivity after pregnancy occurs (Groer et al. 2015). But there are other reproductive differences that may explain an increase in immune reactivity in females, such as higher potential rates of infection of the urinary tract. Females tend to acquire many more urinary tract infections than males. This may be partially due to the longer length of males’ urethras (Harrington and Hooton 2000). It is possible that females have adapted a stronger immune system in response to the increase in likelihood of acquiring these infections, although there is no current research investigating this idea.

### Sex Differences in Stress

Responses to stress vary widely between males and females, and this may explain some of the unexpected interactions between inflammation and mental illness reported in both sexes (Handa et al. 1994; Bekhbat and Neigh 2018). Stress can both upregulate and downregulate the immune response, depending on the length of exposure, the type of stress, and the frequency with which a person is exposed to stress (Dhabhar 2003). Stress pathways also closely interact with sex hormones, and inflammation may actually be upregulated or downregulated, depending on the level and type of sex hormone present (Chakrabarti et al. 2008; Corcoran et al. 2010). Studies show higher female cortisol levels in general, but in some studies of children, male and female cortisol levels are similar in the morning and males have higher levels in the afternoon (Cicchetti et al. 2010). Studies investigating acute stress have found that females have stronger cortisol responses compared to males (Cameron et al. 2017), while other studies find the same level of cortisol, but sex differences in their perception of feelings of stress (Helbig and Backhaus 2017). It’s possible that some of these inconsistencies are related to the duration and type of stress. Some have hypothesized that female sex hormones may provide a protective effect when faced with acute stress, but a negative effect when stress becomes chronic (Zoladz et al. 2019).

Females have been shown to experience more severe depressive symptoms than males when treated with interferons, a cytokine that promotes inflammatory anti-viral action, during an infection (Koskinas et al. 2002; Udina et al. 2012). Unfortunately many seemingly contradictory results have been found that muddy the relationship between the immune and neurological systems (Kohler et al. 2014; Eyre et al. 2015).

## Norm of Gender Experience: Introducing the Psycho-Social Stress Theory

**Fig. 1:**
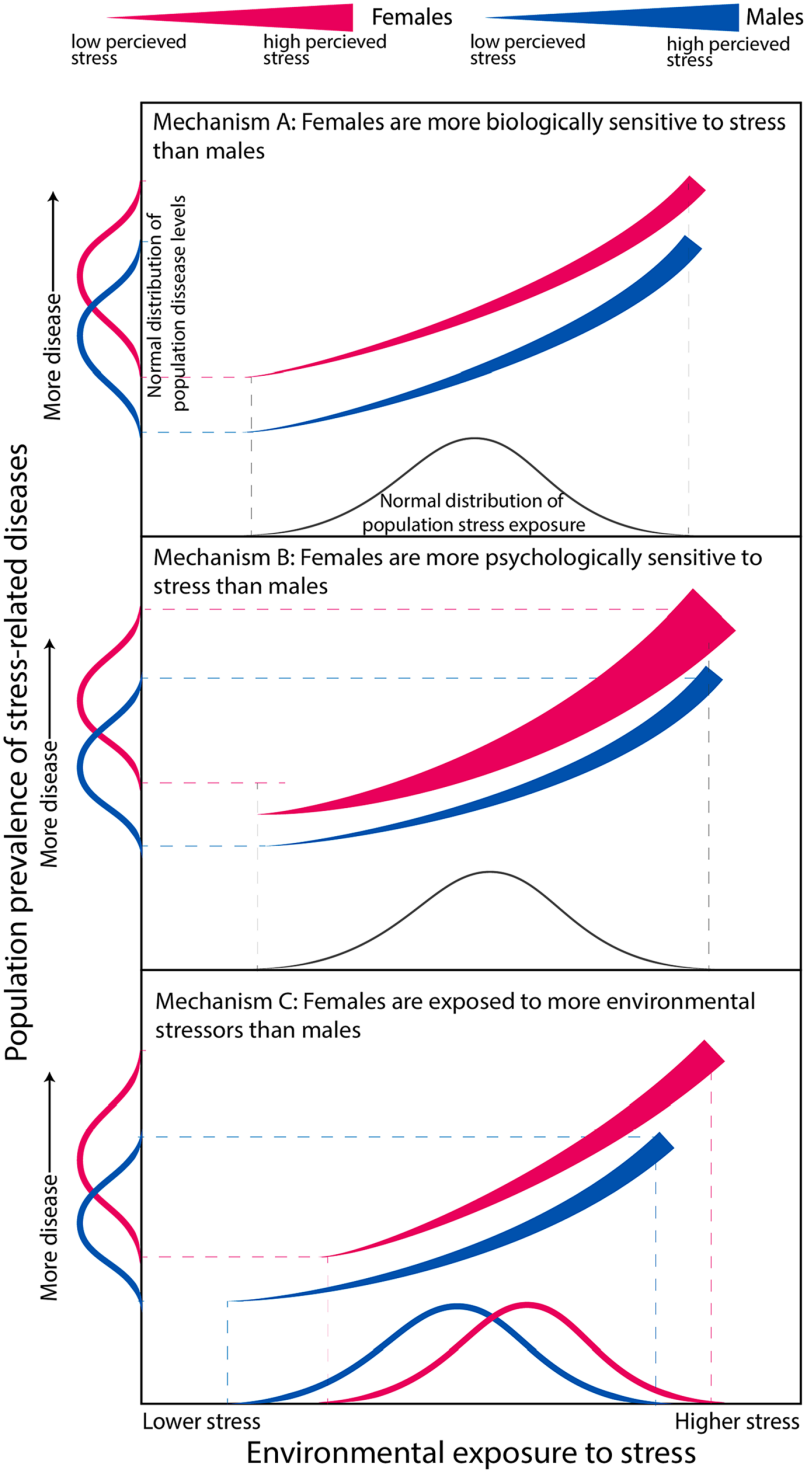
Norm of gender experience: the PSS mechanisms of male and female susceptibility to stress. Pink lines demonstrate hypothetical female trends and blue lines demonstrate hypothetical male trends. Normal distributions are used to demonstrate exposure to stress in a population, as people experience a variety of stress levels, and a variety of disease levels. Likewise, we use normal distributions to represent disease incidence in populations throughout the globe in males and females. The thickness of the line represents the amount of stress a person perceives that they experience, rather than an objective measure of stress exposure (Color figure online)

There are clear cases of high female incidence of certain mental disorders, such as major depressive disorder (MDD), generalized anxiety disorder, post-traumatic stress disorder (PTSD), and others that show role of stress (Vos et al. 2020). It is unknown whether females are more susceptible to stress biologically, they perceive more stress psychologically, and/or if they experience more stress environmentally. We propose these three mechanisms by which sex differences can arise (Fig. 1).

In one mechanism, which we have called sex-related biological differences, females experience the same range and variety of stress as males but are biologically more susceptible to stress (for example, through having a lower hormonal stress-tolerance threshold) than males. This would result in females showing a higher incidence of stress-related diseases and mental disorders (Fig. 1A).

Next is the role of differences in psychological perception*,* wherein females may perceive certain stimuli as being more stressful than males. Here, although the exposure to stress is the same, the perception of said stress is higher; therefore, the biological response to the stress is also higher, heightening the incidence of stress-related disease (Fig. 1B).

Finally, we propose that females may experience higher stress, both in intensity and variety, than males, which gives rise to females showing a higher prevalence of stress-related diseases and mental disorders (Fig. 1C).

These three mechanisms are not exclusive and together make up the Psycho-Social Stress Theory which can be treated as a form of “extended sexual selection”. Factoring in the role of PSS, the grand theory of sexual dimorphism in disease and heath can be called Sexual Selection-Sex Hormone-Psycho-Social Stress Theory (SS-SH-PSS Theory).

## Supporting Evidence for the New Theory

**Fig. 2:**
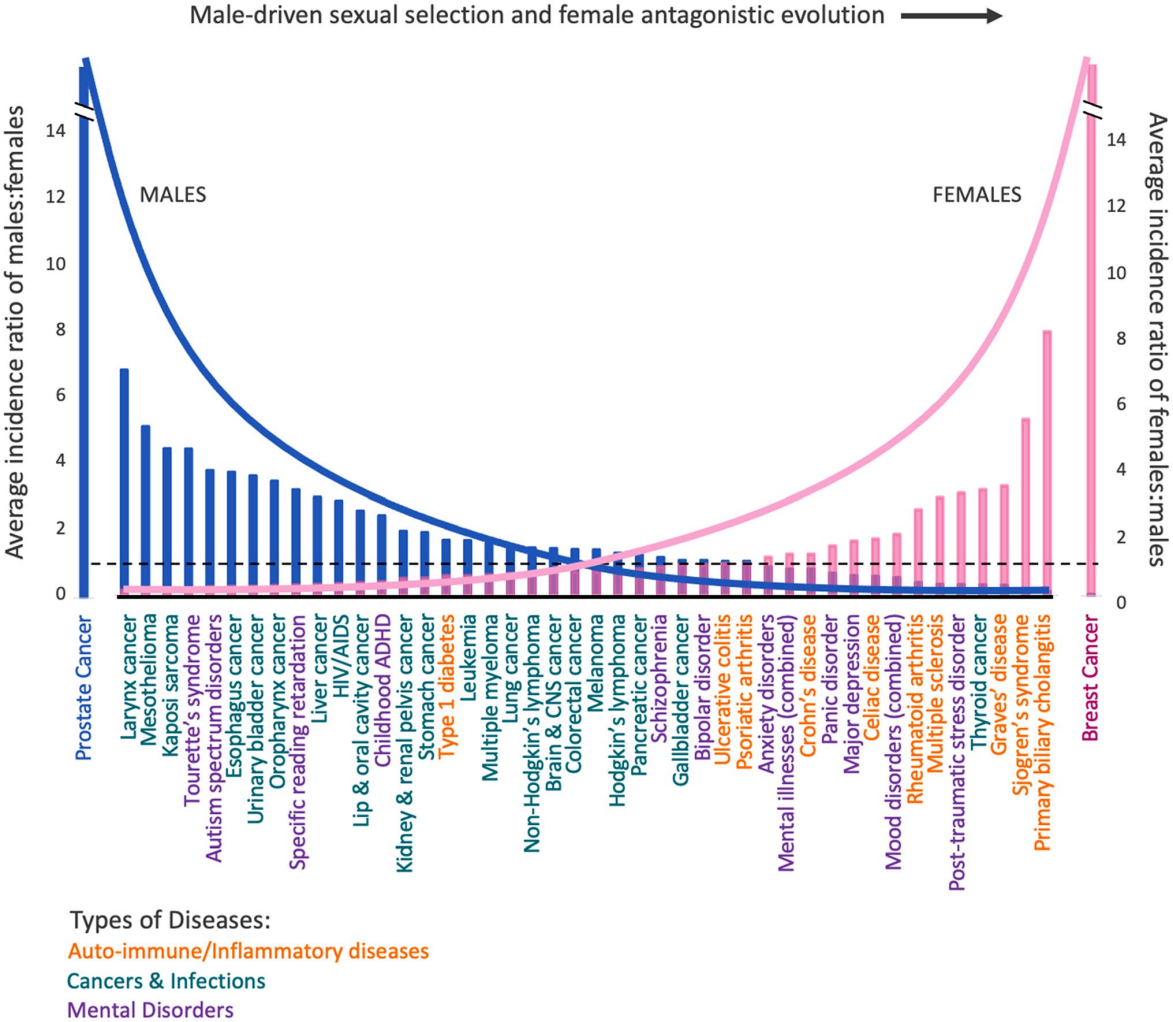
Comparison of the incidence ratios between males and females for many diseases and disorders. Both the male-to-female ratio (blue bars) and the female to male ratio (pink bars) are represented. The theoretical distributions of disease ratios, as predicted by Singh et al. (2021), are represented for the ratios of males to females (blue line) and females to males (pink line). The 1:1 ratio of disease, where both sexes have an equal incidence rate, is indicated by a dotted horizontal line (Color figure online)

We provide supporting evidence for sex-biased diseases and mental disorders from a worldwide survey of disease incidence rates. The estimated number of incidences per year, and the ASIRs (age-standardized incidence rates) for both sexes for each disease, were obtained for various countries to calculate the male-to-female disease incidence ratio (Table 1). Examples of sex-specific diseases (such as breast cancer, corpus uteri cancer, ovarian cancer, cervical cancer, and prostate cancer) are provided in comparison to non-sex-specific diseases. The overall trend of the male-to-female incidence ratio of diseases can be seen in Fig. 2. Overall, this reveals a male-bias with respect to cancers, and a female bias with respect to autoimmune diseases. The neurological diseases and mental disorders are variable, with some more prevalent in males, and others more prevalent in females.

**Table 1:** The sex-bias of disease incidence ratios between males and females

Disease	# of countries	ASIR males	ASIR females	Ratio of males: females	Ratio of females: males
*Sex-specific cancer*					
Breast cancer	13	0.84	91.87	0.01	**109.37**
Cervical cancer	2	0	7.62	0	**N/A**
Ovarian cancer	13	0	9.64	0	**N/A**
Uterine cancer	3	0	29.9	0	**N/A**
Prostate cancer	8	86.85	0	*N/A*	0.00
Testicular cancer	8	6.16	0	*N/A*	0.00
*Non-sex-specific cancer*					
Brain and central nervous system cancer	2	8.47	5.86	*1.45*	0.69
Colorectal cancer	12	52.14	34.09	*1.56*	0.65
Esophageal cancer	12	7.01	1.79	*4.72*	0.26
Hodgkin’s lymphoma	2	3.1	2.29	*1.35*	0.74
Kidney and renal pelvis cancer	14	11.81	5.75	*2.11*	0.49
Laryngeal cancer	2	4.66	0.7	*6.66*	0.15
Leukemia	12	11.28	6.79	*1.63*	0.60
Lip and oral cavity cancer	2	11.26	4.46	*2.69*	0.40
Liver cancer	12	14.05	4.29	*3.2*	0.31
Lung cancer	13	48.33	25.66	*2.15*	0.53
Melanoma	2	42.2	29.15	*1.4*	0.69
Multiple myeloma	2	8.75	5.6	*1.56*	0.64
Non-Hodgkin’s lymphoma	14	15.5	10.06	*1.58*	0.65
Pancreatic cancer	14	10.43	8.17	*1.29*	0.78
Stomach cancer	8	11.54	5.69	*2.06*	0.49
Thyroid cancer	14	4.04	8.62	0.55	**2.13**
Urinary/bladder cancer	2	26.5	7.05	*3.79*	0.27
*Autoimmune/inflammatory disorders*					
Graves’ disease	1	N.D	N.D	0.29	**3.45**
Multiple sclerosis	14	1.63	3.48	0.49	**2.13**
Primary biliary cholangitis	1	N.D	N.D	0.12	**8.33**
Psoriasis	12	153.3	153.74	0.997	**1.003**
Rheumatoid arthritis	15	9.64	25.5	0.38	**2.65**
Sjogren’s syndrome	2	N.D	N.D	0.18	**5.56**
Type 1 diabetes	15	3.1	2.29	*1.35*	0.74
*Neurological disorders and mental illness*					
Anxiety disorders	7	600.4	696.8	0.86	**1.16**
Autism spectrum disorder	8	24.48	6.78	*3.64*	0.28
Bipolar disorder	6	66.85	66.17	*1.02*	0.99
Major depression	7	2489.24	4290.02	0.58	**1.72**
Panic disorder	6	N.D	N.D	0.64	**1.56**
Post-traumatic stress disorder	6	N.D	N.D	0.31	**3.23**
Schizophrenia	6	15.02	13.75	*1.09*	0.92

The age-standardized incidence rate (ASIR) of diseases for sex-specific and non-sex-specific cancers, autoimmune/inflammatory disorders, and neurological disorders and mental illnesses are categorized, and the male-to-female ratio for each disease is specified. Italic cells indicate diseases that are more prevalent in males than females (and therefore their male-to-female ratio is greater than 1), and bold cells show diseases that are more prevalent in females (where the female to male ratio would be greater than 1). Data for this table are from the years 2013–2018 and are acquired from: World Health Organization (WHO), Statistics Canada, Canadian Cancer Society, American Cancer Society, Australian Institute of Health and Welfare, and Australia Cancer Incidence and Mortality (ACIM) Workbooks, Global Health Exchange (GHDx), Sexual Dimorphism in Autoimmune Disease (McCombe et al. 2009), National Institute of Allergy and Infectious Disease (NIH), Global Health Exchange (GHDx), Centers for Disease Control and Prevention (CDC), and the extreme male brain theory of autism (Baron-Cohen)

*Sources of data*: Disease incidence rates for each year, separated by sex, were obtained, and cross-referenced for validity from several main sources, which are described in the supplementary information. Data for this study were obtained from a selection of countries around the globe that have accurate and reliable health databases: Canada, the United States, France, Germany, the United Kingdom, Austria, Belgium, Sweden, Denmark, and Italy, as well as Korea, Japan, and Singapore for certain diseases. Since the data collected were based on limited publicly available data, the year of each disease incidence varied slightly. However, this did not detract from the overall trends demonstrated within this study. Most cancer data presented in this study were obtained through the usage of the WHO database under Cancer Today (a subsection of the Global Cancer Observatory, GCO). Cancer Today provides estimates of the incidence, mortality, and prevalence of 36 different cancer types from a total of 185 different countries and territories. However, given the limited quality and coverage of cancer data, particularly from (but not limited to) low- and middle-income countries, each estimate was interpreted with caution, and the data obtained were cross-referenced to either the official health database of the country or a research publication on the disease. Most of the remaining data were obtained through the GHDx data catalog, created and supported by the Institute for Health Metrics and Evaluation (IHME 2021). GHDx provides information about data from different places and providers in the context of health and demographic research, which proved useful for this study. Some metadata from datasets obtained from research publications used in this study were cross-referenced with GHDx to validate their accuracy. While these data are not original, they can be obtained by writing to the authors. Statistical analysis: male-to-female disease incidence ratios were calculated using the age-standardized incidence rate (ASIR) of each sex in a particular country for a particular year. The ASIR indicated in Table 1 represents the number of new cases of diseases in each country for a given sex, year, and age group, divided by the total infected standard population for that sex, year, and age group, multiplied by 100,000. These are expressed as the number of incidences per 100,000 population. This standardization is necessary and important when comparing disease statistics between multiple populations with different age structures, as age has an important influence on different diseases. Each disease is grouped into one of four categories: sex-specific cancers, non-sex-specific cancers, autoimmune diseases, and mental diseases and disorders. Since the focus of this study was to detect broad trends in sex differences, multi-year data are presented wherever available. Quantitative differences in disease incidence between years within a country and between countries were not considered in this study but are under investigation and will be dealt with in a separate study

In sex-specific diseases, there is a clear sex dominance whereby only one sex is susceptible to the disease [aside from breast cancer, which, in effect, acts like a sex-specific disease (see Table 1)]. These patterns are almost always present in diseases of the reproductive organs, such as the testes or the uterus. The high incidence of cancer in sex organs and tissues is presumably related to the high rate of mutations to be expected, due to repeated cycles of DNA replication (Tomasetti and Vogelstein 2015; Thomas et al. 2016) and lower DNA proof-reading fidelity with increasing age (Yehuda et al. 2001; Neri et al. 2005). Prostate and breast cancers show the highest rates of incidence worldwide (Table 1). The data presented in Table 1 show interesting patterns. First, as much as a two- to three-fold difference is found between cancer rates among countries. Singapore, for example, has the lowest rates of prostate and testicular cancers, and Canada has among the highest rates (Table 1).

### Cancers

Strong sex differences in cancer susceptibility are consistently found in health-related data (Dorak and Karpuzoglu 2012). Males are generally more susceptible to most cancers, having worse overall survival and higher mortality rates, especially in those cancers related to hematologic malignancies (cancers of the blood, bone marrow, and lymphatic system) (Siegel et al. 2017). This finding is further supported through the data presented in this paper (Table 1). Except in the case of thyroid cancer, we found that the male-to-female disease incidence ratio is exceptionally high, especially in the following cancers: urinary bladder cancer, liver cancer, esophageal cancer, lip or oral cavity cancer, laryngeal cancer, and mesothelioma, in which the male ASIR is more than double the ASIR of females (Fig. 2).

Sex hormones, such as testosterone and estrogen, environmental and occupational hazards, such as smoking, a poor diet, or sunlight exposure, are on the list of factors thought to make males more susceptible to cancers (Klein 2000). Several cancers have been linked to sex hormones finding that the presence of female sex hormones provides a protective effect against various cancers like colon cancer (Majek et al. 2013) and stomach cancer (Camargo et al. 2012). Additionally, many current studies focus on specific anatomical differences between sexes as justification for prevalence differences in cancers. Some hypothesize anatomical differences in bladder shape and size, such as a thicker detrusor muscle in males, may be a factor in sex disparity of cases (Mungan et al. 2000).

**Fig. 3:**
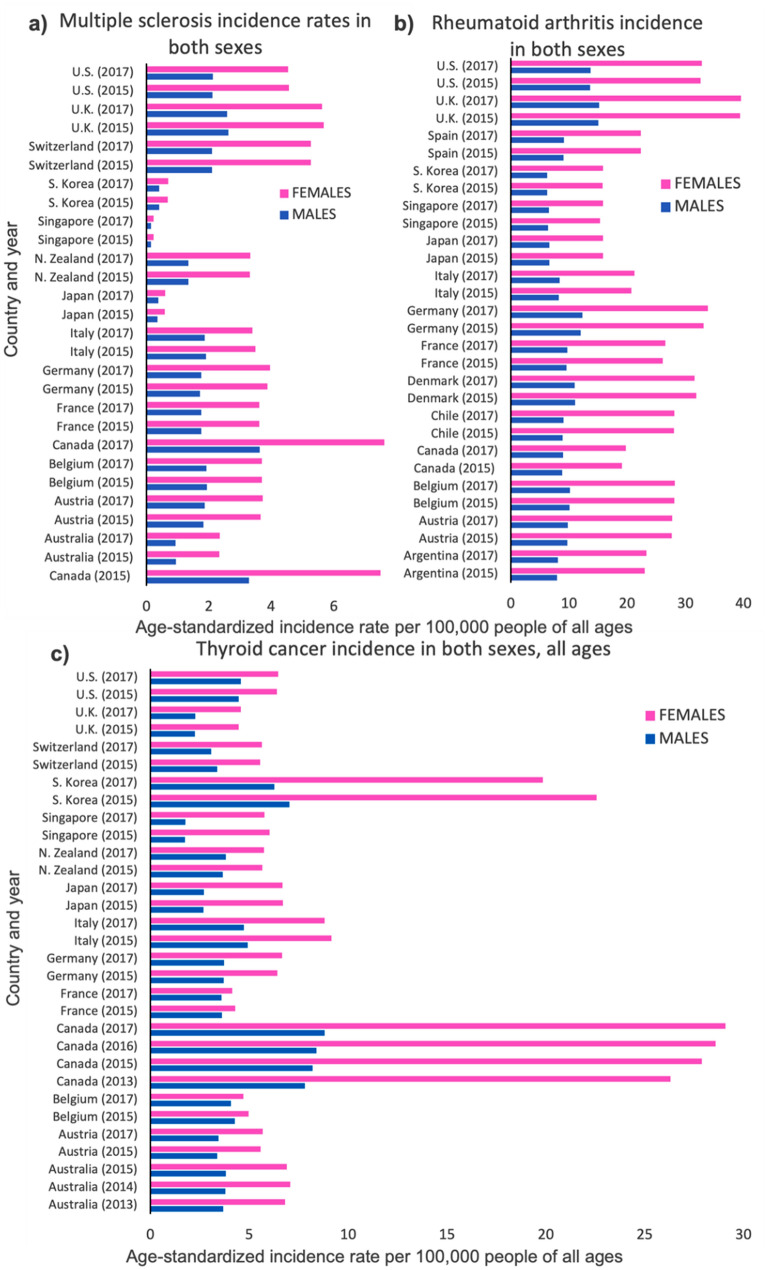
Inflammatory illnesses with higher incidence in females in age-standardized incidence rates/100,000 people of all ages. Multiple sclerosis (**a**), rheumatoid arthritis (**b**), and thyroid cancer (**c**) are displayed with both male (blue) and female (pink) data (Color figure online)

However, a significant female bias is seen in thyroid cancer, in which the female ASIR is approximately double that of the male ASIR (Fig. 3c). Females have, on average, higher levels of thyroid-stimulating hormone (TSH) (Rahbari et al. 2010). It has been hypothesized that the sex disparity in thyroid cancer may be related to sex hormone receptors, especially the estrogen receptor but there isn’t a clear explanation of how this happens. Some hormone-related events greatly increase the risk of thyroid cancer, like hysterectomy and later age pregnancies, but others have seemingly no effect (Caini et al. 2015). For all cancers and countries shown here, except for thyroid cancer, the pattern of sex bias is uniformly male-biased (Table 1).

### Autoimmune Diseases

Unlike most other diseases, there seems to be a clear female bias in terms of autoimmune diseases, whereby females are generally more susceptible than males (Jacobson et al. 1997; Eaton et al. 2007). In our survey data, females show higher ASIRs for various autoimmune diseases, such as multiple sclerosis, rheumatoid arthritis, Graves’ disease, Sjogren’s syndrome, and primary biliary cholangitis (Table 1). Primary biliary cholangitis and Sjogren’s syndrome show the highest incidence rates in females (Fig. 2). However, there is a higher male ASIR for Type 1 diabetes and approximately equal male and female ASIRs for psoriasis.

Sex differences in the efficiency of immune surveillance and genome surveillance mechanisms may lead to the higher female incidence ratio in autoimmune diseases (Dorak and Karpuzoglu 2012). Females seem to show an increase in immune reactivity (Kelly et al. 2018). This may be because they have evolved to exhibit a stronger inflammatory response. Although this response gives females a greater resilience when facing most diseases, it is also possible that their greater immune reactivity makes them more prone to developing autoimmune diseases (Shames 2002; Voskuhl 2011; Ngo et al. 2014). It is also hypothesized that sex-related differences in behavior and organ vulnerability contribute to the dichotomy witnessed in autoimmune disease susceptibility between the sexes (Dorak and Karpuzoglu 2012). This raises the question of whether this phenomenon in females resulted from sexual conflict and antagonistic evolution that led to increased fitness but at the cost of increased susceptibility to autoimmune diseases.

There is no consensus to the reasons behind the increase in male prevalence of type I diabetes, but studies have found links between a gene on the X chromosome and one category (HLA-DR3) of this disease (Cucca et al. 1998). Considering males are more susceptible to acquiring recessive X-linked diseases with only one copy of the X chromosome, this allele may explain the unexpected male-bias in type I diabetes.

## Mental Disorders

**Fig. 4:**
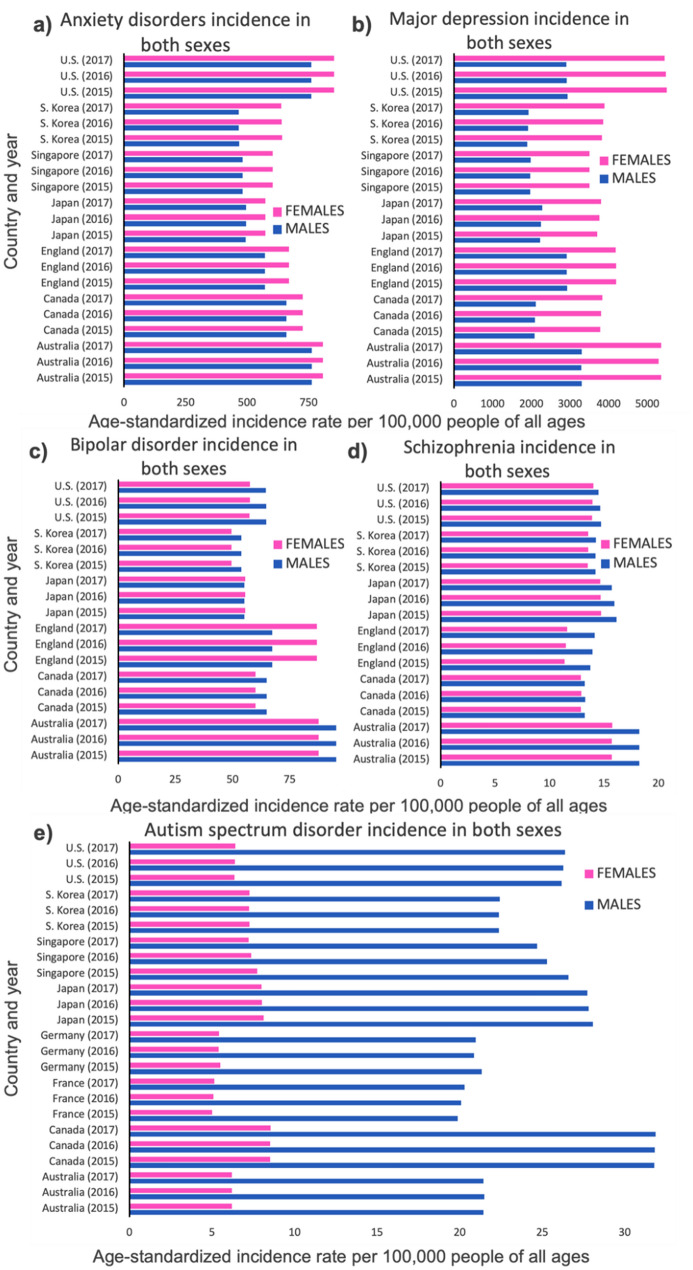
Comparison of male and female levels of mental disorders throughout the world in age-standardized incidence rates/100,000 people of all ages. Any anxiety disorder (**a**), major depressive disorder (**b**), bipolar disorder (**c**), schizophrenia (**d**), and autism spectrum disorder (**e**) are shown in males (blue) and females (pink) (Color figure online)

Males are shown to have higher ASIRs of autism spectrum disorders and schizophrenia while females are shown to have higher ASIRs of depressive and anxiety disorders (Fig. 4). Specifically, post-traumatic stress disorder (PTSD) and panic disorder are both more prevalent in females than in males (Table 1). In addition, the ASIRs for males and females are approximately equal for bipolar disorder.

Mental illnesses and neurological disorders have been strongly connected to inflammation (Kaplan et al. 2015; Makkar et al. 2018; Firth et al. 2019) and experiments promoting immune reactions in model organisms can create behavioral changes that mimic what we identify as depression and anxiety (Konsman et al. 2002; Dantzer et al. 2008; Dantzer 2009). As we discussed earlier, immune responses differ between males and females; therefore, the sex differences exhibited in various mental illnesses are likely at least partially related to differences in inflammatory responses.

There are many points in which sex differences could lead to these differences in mental disorders. The higher rates of mental illnesses in females could be rooted in innate biological differences in neurochemistry, and they could be a result of societal gender roles that generally regard masculinity as being stoic and emotionless (Wisdom et al. 2007; Chonody and Siebert 2008), differences in sex hormones like testosterone and estrogen (Swaab 2003), and the interaction of all of these with the HPA axis. We believe the most plausible explanation for the sex differences seen in these diseases would be that the sexually antagonistic interactions of reproductive conflicts cause differences between the sexes or prompt sexual dimorphism (Bonduriansky et al. 2008), leading to a divergence in stress-response behavior in both sexes through all of the above pathways.

Unfortunately, some physiological findings muddy the waters of the relationship between sex differences in immune responses to stress. Female subjects with depression show greater negative effects of inflammation than males (Derry et al. 2015), but many of the markers of inflammation that are most strongly correlated with mood disorders are significantly higher in males with anxiety and depression than in females (Liukkonen et al. 2011). Often, studies investigating biomarkers of depression find significant differences between depressed and control males, but no difference between depressed and control females (Ramsey et al. 2016). This pattern isn’t just specific to depression. A genome-wide association study (GWAS) of people with generalized anxiety disorder (GAD) found no difference on gene expression between anxious and control women but over 630 differently expressed genes were found in men (Wingo and Gibson 2015). The reasons behind this pattern are unknown.

Additionally, schizophrenia shows a counter-intuitive male-bias. This mental illness has long been linked to heightened levels of inflammation, so we would predict a higher female-bias, but this isn’t what has been found. Schizophrenia is consistently higher in males and females until much later in life (Dunleavy et al. 2022). While no concrete answers have been discovered yet, some studies show interesting findings that demonstrate interactions between sex and schizophrenia that may help uncover this discrepancy. Firstly, treatment of patients with estradiol has shown to reduce psychopathology in both males and females (Kulkarni et al. 2011). Additionally, findings suggest that lower levels of estrogen in women, such as during lower-estrogen phases of the menstrual cycle and post-menopause, are associated with onset and heightened severity of symptoms (Grigoriadis and Seeman 2002).

In summary, our results show that males generally have a higher prevalence of cancers, and females have a higher prevalence of autoimmune and inflammatory diseases. Mental disorders, on the other hand, show an interesting dichotomy. The incidence rates of some mental disorders, such as autism, are higher in males, while those of others, such as major depressive disorder, are higher in females; however, some of the mental disorders reported in females show a high susceptibility to sex hormones and to social stress conditions.

### Gender and Health

In this paper, we reviewed the interactions between sex, disease, and stress, but the topic of gender as a social category must also be discussed. Sex refers to the biological classification of a person’s anatomy and genes, usually into either ‘male’ or ‘female’, although exceptions do exist. Gender, on the other hand, refers to a set of cultural norms, mores, and roles with which an individual identifies that may impact their perception of their surroundings, and their community’s perception of them. The vast majority of disease research treats sex and gender as synonymous, ignoring the distinction between societal roles and biological sex differences in terms of health. This limits our capacity to differentiate between patterns that are influenced by gender-related factors, like abuse or oppression, and sex-related factors, like sex hormones and genetics. We also know that genders are unique in different cultures, whereas sex remains a relative constant. Some cultures routinely recognize more than two genders, most being patriarchal, and a few, matriarchal. Such differences influence the environmental and interpersonal interactions of people in these societies, likely contributing to those people’s health. Because recognition of sex and gender as different concepts is relatively new to the field of science, much existing research uses these terms interchangeably. In this paper, we used the terms that each study uses to classify their participants when referring to external research, and we primarily referred to sex in our theory. That being said, gender may be considered a more important societal predictor of stress than sex, so we may refer to gender occasionally in this regard. Sex-gender is an expanding universe of psychological perceptions of self and others and is likely to play an increasing role in shaping our health.

## Limitations

Currently available data on sex-biased diseases, especially mental disorders, can suffer from gender-biased diagnosis. Historically, research conducted on mental illnesses were biased by gender (Ussher 2013). This has led to some diseases being researched, classified, and diagnosed as male-centric or female-centric. For example, today, autism spectrum disorder has much higher prevalence in males than females but research throughout the nineteenth and twentieth centuries on autism almost exclusively evaluated males. This has resulted in current diagnostic criteria reflecting this male presentation of the disease. Recently, the rate of autism diagnosis in women has increased dramatically as research began to uncover the gender differences that cause differences in presentation between males and females. This problem is not exclusive to autism but represents all diseases without a physical diagnostic tool (e.g., an x-ray of a broken bone), especially when they are so connected to societal gender stereotypes like emotionality and aggression. Research shows that gender bias exists in psychiatric diagnosis (Caplan and Cosgrove 2004; Ussher 2013; Garb 2021). This likely occurs from a combination of three levels, bias of the diagnostic criteria, bias from evaluation tools, and bias from clinician assessing the patient. Some mental illnesses are more prone to gender bias than others. For example, when given a case study with the patients’ genders modulated but all of the same symptoms, diagnostic clinicians were more likely to diagnose females with histrionic personality disorder and males with anti-social personality disorder (Caplan and Cosgrove 2004). Additionally, autism spectrum disorder, attention deficit hyperactivity disorder, and conduct disorder all show gender bias (Garb 2021). Anxiety and depressive disorders did not appear to have significant gender bias meaning that although they are more prevalent in females, males with the same symptoms are equally as likely to receive the same diagnosis (Vanderminden and Esala 2019). Notably, this only addresses one level of bias, as diagnostic criteria still likely have some level of bias to them. Although some level of bias is likely present, we believe that the vast differences in disease prevalence rates that occur cross-culturally demonstrate, at least on the face value, that there are more contributing factors than solely gender bias in diagnosis.

We also note the possibility of some mechanisms of our theory acting more than others. It is possible that differences in experiences of stress (mechanism 3) could account for all differences in female-biased disease rate through gender-based violence and oppression, rendering mechanisms 1 and 2 moot. We don’t doubt that PSS is experienced differently in women and men, as patriarchal societies result in different gender treatments. That being said, the trend of female-biased diseases doesn’t disappear or greatly drop in locations where gender-based violence is less common and/or less overt. Even as societal understandings of femininity differ from location to location, clear sex biases are seen in global rates of many diseases. For this reason, we don’t believe that mechanism 3 is sufficient to explain all of the differences in sex-biased diseases and why mechanisms 1 and 2 are also necessary.

Additionally, we recognize that some research points to the effects of traditionally ‘masculine’ behaviour (e.g., wielding power) as increasing levels of testosterone (van Anders et al. 2015). This could influence the different responses to stress by sex and/or directly impact immune responses, as testosterone is widely thought to have immunosuppressive effects. We don’t believe this works against our theory, but instead runs parallel to it as another mechanism of how sexual selection may have pushed for a stronger immune system in females.

Finally, much of the data we collected was from higher income countries as they have publicly available data on rates of disease. This presents a bias as smaller and lower-income countries were not evaluated. Although cultural stigma, complexity of circulating inflammatory cytokines, and wealth, complicate the story, we believe that our addition to the SS-SH Theory to create the SS-SH-PSS Theory better explains the reality of sex-biased diseases, especially mental illnesses. We strongly encourage the further exploration of the relationships between exposure to stress and female-biased diseases, while working to determine means to reduce gender biased diagnostic practices, to continue to uncover the complicated phenomena between stress, disease, and sex.

## Conclusion

Just as the genomics revolution and the promise of personalized medicine opened the door of genomic complexity, the consideration of sex-biased diseases is opening the door of the “sexualized genome” and the evolution of gender-related health (Singh et al. 2021). A few examples will suffice. First, past deterministic approaches to practicing medicine based on the patients’ biology (and their exposure to their environment) are slowly giving in to consideration of the roles of gender, genealogy, and evolutionary biology. Second, ethnic diversity is adding another dimension to both personalized and gender-related health. The joint consideration of evolutionary biology and molecular medicine is opening a window into the past to see, for example, how sexual selection and patriarchy in the past have shaped women’s health. Third, the science of genomics is showing that ultimate, or evolutionary, and proximal, or functional, causes are not mutually exclusive, competing hypotheses; instead, the former is embedded in the latter, and they are two ends (back and front) of the same evolutionary spectrum. This extended framework can provide medicine with the power of prediction. Fourth, gene–environment interaction is creating a new dimension in gender-related health by incorporating psycho-social factors. Finally, in all this progress, social stress is emerging as a major determinant of female health.

This paper provides an evolutionary framework for what has been known for some time: that males are more susceptible to various cancers, infections, and certain mental disorders, while females predominantly have higher incidence rates of thyroid cancer, autoimmune diseases and other mental disorders, often pertaining to emotions. We use these results to extend the SS-SH Theory (Singh et al. 2021) to include PSS and make it a grand SS-SH-PSS Theory to explain gender dimorphism in disease and health. PSS can be treated as a form of extended sexual selection.

The toll taken by social stress on women’s health is hidden. The ways that female-specific biological properties and factors influence inflammation and how PSS impacts female susceptibility to diseases and mental disorders should be studied to shed light on potential female-specific treatment methods. Finally, diseases affected by social stress conditions, such as mental disorders, cannot be treated in terms of “all or none”; they are likely to represent a continuously distributed disease liability with individual thresholds. Both the types of liability and the thresholds are likely to vary as a function of age and social conditions, making a larger section of the female population at risk of problems than it appears to be on the surface.
